# Co-expression and prognostic significance of the HER family members, EGFRvIII, c-MET, CD44 in patients with ovarian cancer

**DOI:** 10.18632/oncotarget.24791

**Published:** 2018-04-13

**Authors:** Soozana Puvanenthiran, Sharadah Essapen, Ben Haagsma, Izhar Bagwan, Margaret Green, Said Abdullah Khelwatty, Alan Seddon, Helmout Modjtahedi

**Affiliations:** ^1^ School of Life Sciences, Pharmacy and Chemistry, Kingston University London, Kingston, UK; ^2^ St Luke's Cancer Centre, Royal Surrey County Hospital, Guildford, UK; ^3^ Department of Histopathology, Royal Surrey County Hospital, Guildford, UK

**Keywords:** ovarian cancer, HER family members, c-MET, CD44, prognosis

## Abstract

EGFR and HER-2 are important targets but none of the monoclonal antibodies or small molecule tyrosine kinase inhibitors specific for the HER members has been approved for the treatment of patients with ovarian cancers. In some studies, co-expression of other growth factor receptors has been associated with resistance to therapy with the HER inhibitors. The aim of the present study was to determine the relative expression, cellular location, and prognostic significance of HER-family members, the EGFR mutant (EGFRvIII) c-MET, IGF-1R and the cancer stem cell biomarker CD44 in 60 patients with FIGO stage III and IV ovarian cancer. At cut off >5% of tumour cells with positive staining, 62%, 59%, 65% and 45% of the cases were EGFR, HER-2, HER-3 and HER-4 positive, and 3%, 22% and 48.3% of the cases were positive for EGFRvIII, c-MET, and CD44 respectively. Interestingly, 23% co-expressed all four members of the HER family. On univariate analysis, only EGFR staining at >50% of tumour cells (HR = 3.57, *p* = 0.038) and CD44 staining at 3+ intensity (HR = 7.99, *p* = 0.004) were associated with a poorer overall survival. EGFR expression (HR = 2.83, *p* = 0.019) and its co-expression with HER-2, HER-3, HER-2/HER-3, and c-MET were all associated with poorer disease-free survival. Our results suggest co-expression of the HER-family members is common in Stage III and IV ovarian cancer patients. Further studies on the prognostic significance and predictive value of all HER family member proteins for the response to treatment with various forms of the HER inhibitors are warranted.

## INTRODUCTION

Despite major advances in our understanding of cancer biology and pathogenesis, cytoreductive surgery, platinum-based chemotherapy and targeted therapy, ovarian cancer remains as one of the world's most aggressive and lethal types of gynecological cancer [1–6]. Worldwide, an estimated 238,700 women were diagnosed with ovarian cancer, and 152,000 died of the disease in 2012 [7]. As there are currently no reliable screening methods and the early stages of the disease usually present no obvious symptoms, the great majority of ovarian cancer patients are diagnosed at advanced stages of the disease [8, 9]. While the five-year survival rate for ovarian cancer patients diagnosed at stage I and II of the disease is approximately 90% and 65% respectively, it reduces to around 33% and 18% for patients diagnosed at stage III and stage IV respectively [10, 11]. Moreover, in spite of initial chemo-sensitivity, most ovarian cancers acquire a drug-resistant phenotype, which in turn makes it largely a recurrent and incurable disease [6, 9, 12, 13]. These statistics highlight the urgent need for the identification of marker(s), which are important in the progression of ovarian cancer, for use in the early detection of the disease, and for guiding treatment [14]. It is also important to develop novel, more effective and less toxic therapies for patients with ovarian cancer [9, 13, 15–17].

In the past three decades, increased expression or activation of the epidermal growth factor receptor (EGFR) family (also called ErbB/HER family) have been reported in a wide range of epithelial cancers, and in some studies have also been associated with a poorer prognosis and resistance to therapeutic options [18–22]. The HER family members includes, EGFR, HER-2, HER-3 and HER-4. Homodimersiation and heterodimerisation of the HER family members, as a result of ligand binding and or receptor mutation, results in the activation of several downstream cell signaling pathways and ultimately tumour cell proliferation, reduced apoptosis, tumour migration and invasion, as well as resistance to therapy [19, 22, 23]. To date, several types of HER inhibitors have been approved for the treatment of patients with a wide range of epithelial cancers including the anti-EGFR monoclonal antibodies (mAbs) cetuximab, panitumumab, necitumumab and nimotuzumab, anti-HER mAbs trastuzumab, pertuzumab and ado-trastuzumab emtansine, and small molecule tyrosine kinase inhibitors (TKIs) such as EGFR specific erlotinib and gefitinib, dual EGFR/HER-2 TKI lapatinib, and pan HER-family TKIs afatinib and neratinib [24–26]. Despite these advances, many patients simply do not respond or eventually develop resistance to therapy with the EGFR inhibitors, and none of the HER inhibitors have yet been approved for the treatment of ovarian cancer patients [27–34]. In some studies, tumour heterogeneity, expression of other members of the HER family (e.g. HER-3), mutation of a HER family member (e.g. the EGFRvIII), the co-expression of other heterologous growth factor receptors (e.g. c-MET, IGF-1R), and the presence of cancer stem cells has been suggested as possible mechanisms of resistance to therapy with the HER inhibitors and cytotoxic drugs. These together with poor patient selection may therefore have contributed to the disappointing clinical trials with the HER inhibitors in ovarian cancer [35–43].

We have recently studied the impact of the growth factor receptor expression and the putative ovarian cancer stem cell marker CD44 on the sensitivity of a large panel of human ovarian cancer cells to treatment with various forms of HER-TKIs (Gefitinib, Erlotinib, Lapatinib, Sapitinib, Afatinib, Canertinib, Neratinib) and other TKIs including crizotinib (C-met/Alk inhibitor), NVP-AEW541 (IGF-1R inhibitor), dasatinib (v-abl/src/c-KIT TKI) and imatinib (v-abl/c-KIT/PDGFR TKI) [33]. Of the HER inhibitors, the irreversible pan-HER TKIs were found to be the most effective for inhibiting the growth and migration of ovarian cancer cells. However, to our knowledge, there is currently no comprehensive study on the relative expression, cellular location and prognostic significance of all members of the HER family, c-MET, IGF-1R, and the putative ovarian cancer stem cell biomarker in patients with ovarian cancer. Therefore, in this study we examined the relative expression and cellular location of all members of the HER family, the type-III mutated form of EGFR (EGFRvIII), c-MET, IGF-1R and CD44 by immunohistochemistry in patients with FIGO stage III and IV ovarian cancer and their associations with clinico-pathological parameters, overall survival and disease-free survival.

## RESULTS

### Clinicopathological characteristics

Patient clinicopathological characteristics are summarised in Table 1. The mean overall survival in this study was 2.62 ± 1.7 years (median 2.3 years), and the mean disease-free survival was 25.2 ± 20 months (median 18 months). No association was found with the clinicopathological characteristics and overall survival of these patients. However, overall survival was found to be poorer in the eight patients who received bevacizumab (*p* = 0.021, Table 1)

**Table 1:** Clinicopathological characteristics and overall survival of FIGO stage III and IV ovarian cancer patients

Characteristics	Number of patients (%)	Overall survival in years (mean ± SE)	*p*- value	Disease free survival in months (mean ± SE)	*p*- value
Age in years					NS
≤ 65	22 (36.7)	4.6 ± 0.2	NS	31.2 ± 5.7	
> 65	38 (63.3)	4.8 ± 0.2		46.3 ± 5.1	
Subtypes					NS
Serous	51 (85)	4.7 ± 0.2	NS	42.8 ± 4.2	
Non-serous	9 (15)	5.1 ± 0.4		23.0 ± 9.2	
FIGO stage					NS
III	44 (73.3)	4.8 ± 0.3	NS	42.1 ± 4.8	
IV	16 (26.7)	4.5 ± 0.1		35.1 ± 5.3	
Grade					NS
G2	2 (3.3)	NS	NS	20.0 ± 0.0	
G3	58 (96.7)			41.2 ± 4.1	
Bevacizumab treated^*^					NS
Yes	8 (13.3)	4.3 ± 0.1	0.021	45.4 ± 6.5	
No	49 (81.7)	5.0 ± 0.2		37.7 ± 4.6	

NS: not significant (*P* > 0.05),

^*^data for bevacizumab treated missing in 3 patients. OS and DFS analysis was conducted by omitting the missing data.

Overall survival and disease free survival relative to the indicated features was determined by Kaplan-Meier analysis and the log-rank test. *P*-value of < 0.05 was considered significant.

### Most ovarian cancer cases were HER positive but EGFRvIII negative by immunohistochemistry

**Figure 1:**
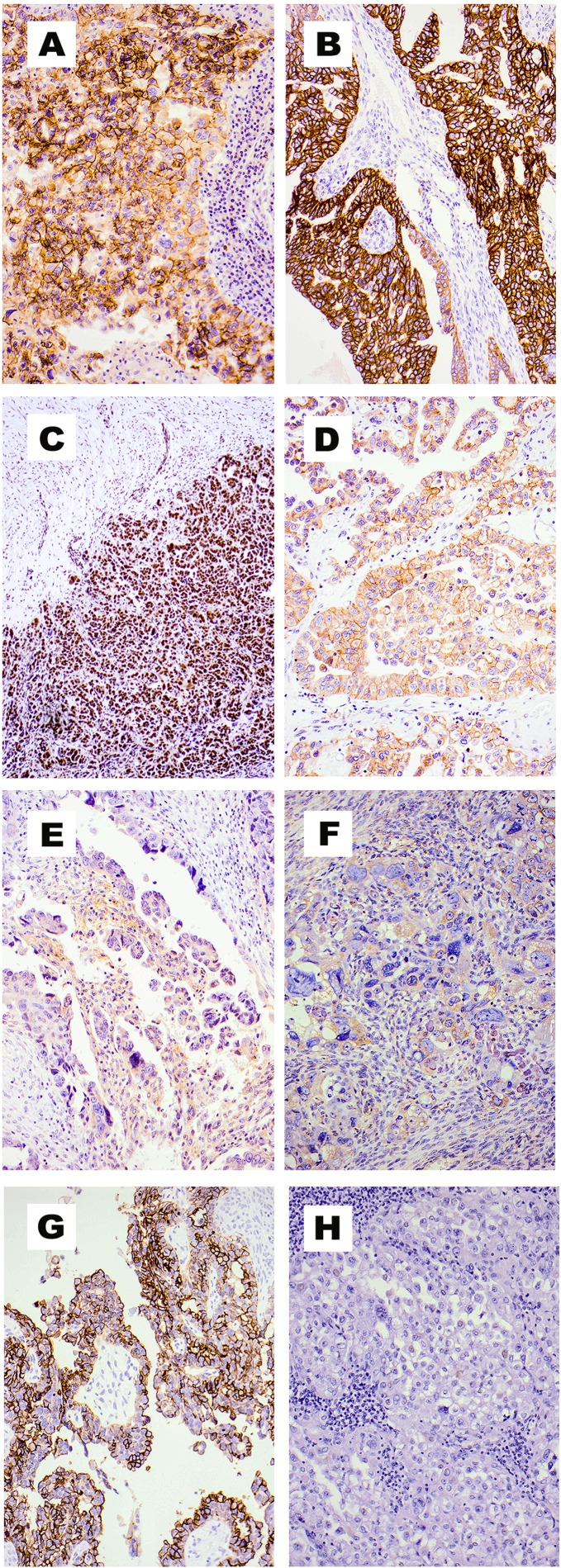
Immunohistochemical staining of tumour specimens from patients with stage III and IV ovarian cancer EGFR 2+/3+ membranous (**A**), HER-2 2+ membranous (**B**), HER-3 3+ nuclear (**C**) HER-4 2+ membranous (**D**) EGFRvIII 1+ cytoplasmic (**E**), c-MET 2+ cytoplasmic (**F**), CD44 3+ membranous (**G**), and negative control (**H**). Magnification x100.

The expression pattern of EGFR, HER-2, HER-3 and HER-4 was determined in 60 FIGO stage III and IV ovarian tumour specimens. At a cut-off value of above 5% of tumour cells with positive immunostaining, the expression of EGFR was seen in 37/60 (61.7%) ovarian cancer cases (Table 2). Of these, the cellular location of EGFR staining was membranous and cytoplasmic in 20/60 (33.3%), and 17/60 (28.3%) of the cases examined respectively, and the intensity of EGFR staining of 2+ or 3+ was present in five patients (Table 2, Figure 1). The great majority of tumour specimens (93%) were HER-2 positive at the cut-off value of above 5%. However, in contrast to EGFR, the cellular location of HER-2 immunostaining was predominantly cytoplasmic (50/60, 83.3%) (Table 2, Figure 1). Interestingly, using the RTJ.2 mAb the cellular location of HER-3 was mostly nuclear and of these, at cut-off value of above 5% of tumour cells with positive immunostaining was detected in 39/60 (65%) of the cases examined (Table 2, Figure 1). At the same cut-off value of above 5% of tumour cells with positive immunostaining, HER-4 expression was detected in 27/60 (45%) of the cases examined, with the predominant location of immunostaining being cytoplasmic (43%) (Table 2, Figure 1). Finally, the expression of EGFRvIII was rare in tumour specimens from these patients and only weak cytoplasmic staining was seen in 3.3% (2/60) of the ovarian cancer cases examined (Table 2, Figure 1). The percentage of positive tumour specimens, at other cut-off values (i.e. above 10%, 20% or 50% of the tumour cells with positive immunostaining) are summarised and presented in Table 2.

**Table 2:** Expression of HER family members, c-MET, IGF-IR and CD44 determined by immunohistochemistry in FIGO stage III and IV ovarian cancer patients, data are presented based on the percentage of tumour cells with positive staining, the intensity of staining and the cellular location of staining

No. of positive tumours (%)							
Scoring criteria	EGFR	HER-2	HER-3	HER-4	EGFRvIII	c-MET	CD44
Percentage of positive tumour cells (%)							
**>5**	37 (61.7)	56 (93.3)	39 (65)	27 (45)	2 (3.3)	13 (21.7)	29 (48.3)
**>10**	29 (48.3)	55 (91.7)	37 (61.7)	17 (28.3)	0	7 (11.7)	11 (18.3)
**>20**	25 (41.7)	50 (83.3)	33 (55)	10 (16.7)	0	7 (11.7)	10 (16.7)
**>50**	12 (20)	44 (73.3)	18 (30)	3 (5)	0	5 (8.3)	4 (6.7)
Intensity							
**1+**	32 (53.2)	19 (31.7)	28 (46.7)	25 (41.7)	2 (3.3)	9 (15)	7 (11.7)
**2+**	4 (6.7)	25 (41.7)	19 (31.7)	2 (3.3)	0	4 (6.7)	13 (21.7)
**3+**	1 (1.7)	12 (20)	5 (8.3)	1 (1.7)	0	0	10 (16.7)
Sub-cellular localisation							
**Membranous**	20 (33.3)	6 (10)	0	2 (3.3)	0	0	30 (50)
**Cytoplasmic**	17 (28.3)	50 (83.3)	0	26 (43.3)	2 (3.3)	13 (21.7)	0
**Nuclear**	0	0	40 (66.7)	0	0	0	0

All tumours were IGF-IR negative

### Expression level of IGF-1R, c-MET and CD44 determined by immunohistochemistry

Next, we examined the expression pattern of other growth factor receptors (c-MET and IGF-1R) and one of the putative ovarian cancer stem cell markers, CD44, in tumour specimens from these patients. Interestingly, while the anti-IGF-1R Clone 24–31 mAb stained the IGF-1R positive control placenta, none of the 60 ovarian cancer specimens were IGF-1R positive (Table 2, Figure 1). At the cut of value of above 5% of the tumour cells with positive immunostaining, the expression of c-MET was detected in 13/60 ovarian cancer cases (21.7%) with its cellular location all being cytoplasmic (Table 2, Figure 1). The cellular location of CD44 staining was only membranous. At cut-off value of above 5% of tumour cells with positive immunostaining, 48% of the ovarian cancer cases were CD44 positive (Table 2, Figure 1). The percentage of positive cases at other cut-off values and based on the intensity of immunostaining for these biomarkers are summarised in Table 2.

### Co-expression of different members of the HER family in patients with ovarian cancer

While various studies have examined and reported the expression level of the individual members of the HER family, the number of studies on the co-expression of these receptors are limited. Therefore, next the co-expression of HER family members, c-MET, and CD44 were analysed, and the results are summarised in Table 3. For example, at cut of values of above 5% of tumour cells with positive staining, dual expression of EGFR/HER-2, EGFR/HER-3 and EGFR/HER-4 were present at 62%, 45%, and 28% of the cases examined respectively (Table 3). In addition, 43%, 23% and 28% of the cases had co-expression of EGFR/HER-2/HER-3, EGFR/HER-3/HER-4 and EGFR/HER-2/HER-4 respectively. Interestingly, of the 60 cases examined, 23% had co-expression of all four members of the HER family (Table 3). In 3% and 12% of the cases examined, the co-expression of all four members of the HER family was also accompanied by expression of c-MET and CD44 respectively (Table 3).

**Table 3:** Co-expression of HER family members, c-MET, and CD44 determined by immunohistochemistry in patients with FIGO stage III and IV ovarian cancer. The percentage was scored based on the cut of value of above 5% of tumour cells with positive staining

Markers	No. of positive tumours (%)	Markers	No. of positive tumours (%)
**EGFR/HER-2**	37 (61.7)	HER-2/HER-4/C-MET	5 (8.3)
**EGFR/ HER-3**	27 (45)	HER-3/HER-4/C-MET	3 (5)
**EGFR/HER-4**	17 (28.3)	EGFR/HER-2/HER-3/C-MET	6 (10)
**EGFR/HER-2/HER-3**	26 (43.3)	EGFR/HER-2/HER-4/C-MET	2 (3.3)
**EGFR/HER-3/HER-4**	14 (23.3)	EGFR/HER-2/HER-3/HER-4/C-MET	2 (3.3)
**EGFR/HER-2/HER-4**	17 (28.3)	EGFR/CD44	17 (28.3)
**EGFR/HER-2/HER-3/HER-4**	14 (23.3)	HER-2/CD44	28 (46.7)
**HER-2/HER-3**	37 (61.7)	HER-3/CD44	17 (28.3)
**HER-2/HER-4**	26 (43.3)	HER-4/CD44	14 (23.3)
**HER-3/HER-4**	19 (31.7)	EGFR/HER-2/CD44	17 (28.3)
**EGFR/C-MET**	8 (13.3)	EGFR/HER-3/CD44	11 (18.3)
**HER-2/C-MET**	13 (21.7)	EGFR/HER-4/CD44	9 (15)
**HER-3/C-MET**	9 (15)	HER-2/HER-3/CD44	17 (28.3)
**HER-4/C-MET**	5 (8.3)	HER-2/HER-4/CD44	13 (21.7)
**EGFR/HER-2/C-MET**	8 (13.3)	HER-3/HER-4/CD44	9 (15)
**EGFR/HER-3/C-MET**	6 (10)	EGFR/HER-2/HER-3/CD44	11 (18.3)
**EGFR/HER-4/C-MET**	2 (3.3)	EGFR/HER-2/HER-4/CD44	7 (11.7)
**HER-2/HER-3/C-MET**	9 (15)	EGFR/HER-2/HER-3/HER-4/CD44	7 (11.7)

### CD44 expression with 3+ intensity is associated with a poorer overall survival

**Figure 2:**
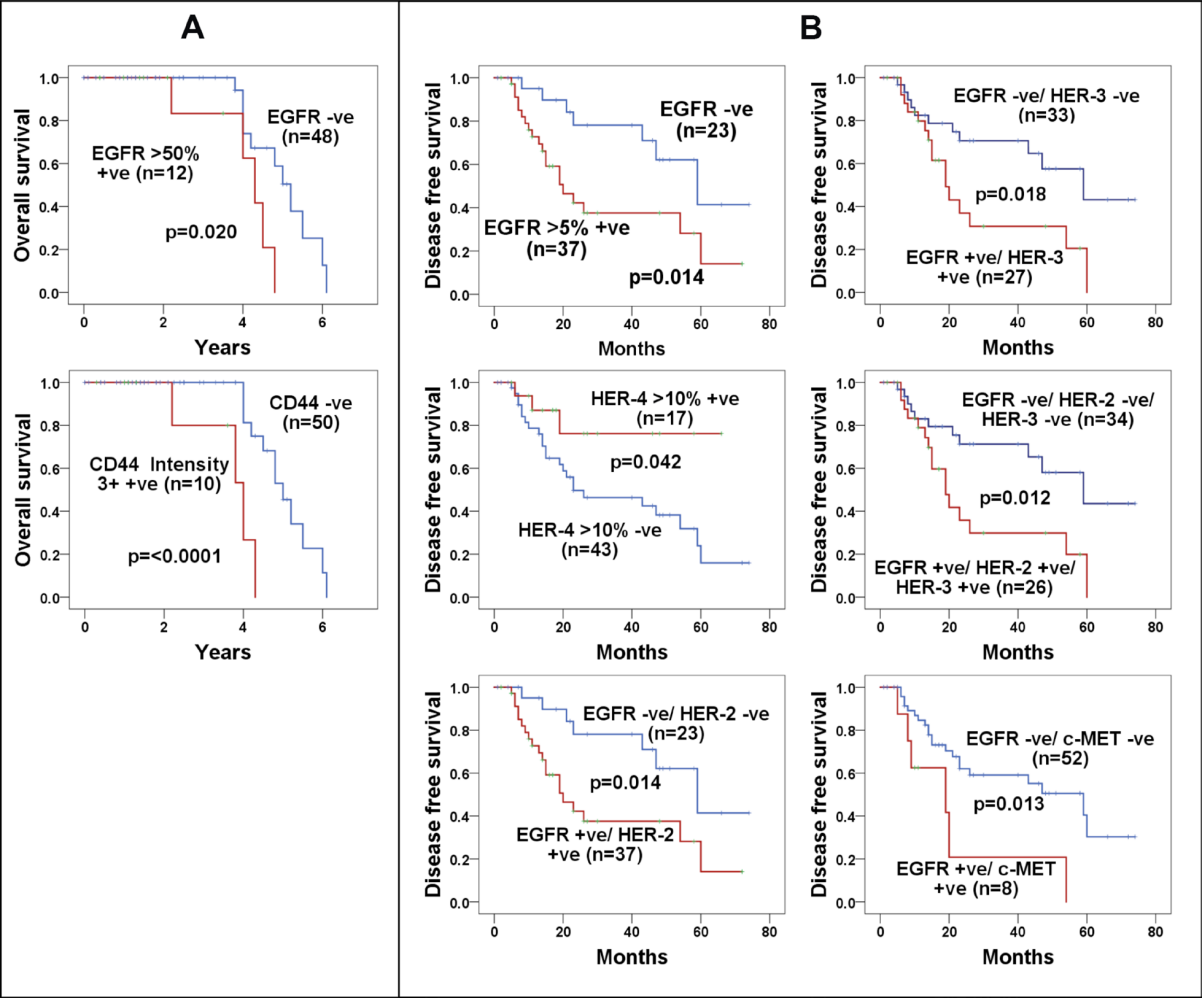
The impact of various biomarker expressions on the overall survival and disease free survival in patients with stages III and IV ovarian cancer (**A**) Kaplan-Meier survival curves of the overall survival for the patients with EGFR staining in >50% of tumour cells, and CD44 staining of 3+ intensity in >5%. (**B**) Kaplan-Meier survival curves of the disease free survival for the patients with total expression of EGFR staining of >5% of tumour cells, HER-4 staining of >10% tumour cells, EGFR & HER-2 co-expression >5% tumour cells, EGFR & HER-3 co-expression >5% tumour cells, EGFR & HER-2 & HER-3 co-expression >5% tumour cells, EGFR & c-MET co-expression of >5% tumour cells. A log-rank test value of P- <0.05 was considered statistically significant.

The association between the expression level of HER family members, at different cut-off values, the intensity of staining and the cellular location of staining and overall survival were determined using the Kaplan-Meier curves and log ranks-test. EGFR expression at >50% of tumour cells with positive EGFR immunostaining was associated with poorer overall survival (*p* = 0.020) (Figure 2A). When using univariate analysis, patients with EGFR expression at cut-off values of >50% had a hazard ratio of 3.6 (CI 1.07 – 11.85 *p* = 0.038, Table 4), however the expression of EGFR >50% did not remain as an independent prognostic factor in multivariate analysis after adjusting for other covariates used in this study (HR 3.8, CI 0.95–15.6, *p* = 0.058, Table 4). No significant association was found between the expression of HER member's at other cut-off values and the overall survival in these patients, and nor between EGFRvIII expression and the overall survival (data not shown).

**Table 4:** Univariate and multivariate analysis of the association between sub-categories of biomarkers in overall survival and the disease free survival

Overall Survival	Univariate			Multivariate		
	Hazard Ratio	95% CI	*P*-value	Hazard Ratio	95% CI	*P*-value
**EGFR >5%**	1.15	0.41–3.21	0.787 (NS)	1.02	0.31–3.31	0.964 (NS)
**HER-2 >5%**	0.61	0.13–2.81	0.531 (NS)	0.41	0.07–2.19	0.298 (NS)
**HER-3 >5%**	0.43	0.16–1.30	0.146 (NS)	0.36	0.16–1.15	0.088 (NS)
**HER-4 >5%**	1.85	0.63–5.37	0.256 (NS)	0.26	0.59–6.55	0.266 (NS)
**c-MET >5%**	1.23	0.33–4.50	0.748 (NS)	1.10	0.29–4.21	0.883 (NS)
**CD44 >5%**	1.08	0.38–3.06	0.882 (NS)	1.12	0.39–3.24	0.823 (NS)
**EGFR >50%**	3.57	1.07–11.85	0.038	3.85	0.95–15.6	0.058 (NS)
**CD44 >5% 3+**	7.99	1.96–32.55	0.004	9.23	1.82–46.7	0.007
Disease free survival						
**EGFR >5%**	2.83	1.18–6.77	0.019	2.53	1.00–6.36	0.048
**HER-2 >5%**	1.73	0.40–7.36	0.455 (NS)	2.44	0.31–6.22	0.660 (NS)
**HER-3 >5%**	0.41	0.61–3.27	0.414 (NS)	1.39	0.64–3.63	0.341 (NS)
**HER-4 >5%**	0.45	0.19–1.07	0.073 (NS)	1.51	0.21–1.27	0.151 (NS)
**c-MET >5%**	1.45	0.61–3.45	0.398 (NS)	1.50	0.62–3.60	0.362 (NS)
**CD44 >5%**	0.87	0.40–1.86	0.721 (NS)	0.95	0.43–2.06	0.896 (NS)
**EGFR >10%**	2.40	1.07–5.37	0.032	2.44	1.06–5.60	0.035
**EGFR + HER-2 >5%**	2.83	1.18–6.77	0.019	2.53	1.00–6.36	0.048
**EGFR + HER-3 >5%**	2.48	1.13–5.42	0.023	2.69	1.15–6.28	0.021
**EGFR + HER-2 +HER-3 >5%**	2.60	1.19–5.67	0.016	2.82	1.22–6.50	0.015
**EGFR + c-MET >5%**	3.05	1.20–7.75	0.019	2.65	0.96–7.31	0.059 (NS)

HR: Hazard ratio; 95% CI: 95% confidence interval; *p*-value < 0.05, NS: non-significant.

*P*-value of < 0.05 was considered significant.

While there was no significant association between CD44 immunostaining at cut-off values >5%, >10% and >20% and overall survival, CD44 immunostaining of 3+ intensity at cut-off values of >5% of tumour cells was associated with poorer overall survival in these patients (3.66 ± 0.39 vs. 5.01 ± 0.20) (*p* <0.0001) (Figure 2A). Using univariate analysis, we found an 8 fold increased risk of poorer overall survival with the expression of CD44 3+ intensity at >5% cut-off value (*p* = 0.004) and this remained an independent prognostic factor for survival in multivariate analysis in this study (*p* = 0.007, Table 4).

### Impact of HER family members, c-MET and CD44 expression on disease-free survival

Of all cut-off values used in this study, only the EGFR positive immunostaining at cut-off values of >5% and >10% of the tumour cells were significantly associated with a poorer disease-free survival (32.34 ± 4.88 vs 53.79 ± 5.78 months, *p* = 0.014 Figure 2B) and (29.64 ± 4.86 vs 47.9 ± 5.05 months, *p* = 0.026, data not shown). There was no significant association between HER-2 positive immunostaining at all cut-off values and disease-free survival in these ovarian cancer cases. However, HER-4 positive immunostaining in >10% of the tumour cells was associated with a better disease-free survival (53.43 ± 6.50 vs. 36.0 ± 4.3 months, *p* = 0.042) in these patients (Figure 2B).

Moreover, there was no significant association between the expression of c-MET alone at all cut-off values (>5%, >10%, >20% and >50%) and disease-free survival. Interestingly, at cut-off values >5% of the tumour cells with positive staining, the co-expression of EGFR/HER-2, EGFR/HER-3, EGFR/c-MET, and EGFR/HER-2/HER-3 were all associated with a poorer disease free-survival in the univariate analysis (Figure 2B, Table 4). Using multivariate analysis, with the exception of EGFR/c-MET co-expression, the co-expression of the HER-family members remained independent prognostic factors of DFS in this study (Table 4).

## DISCUSSION

Ovarian cancer is a leading cause of death from gynaecological cancers [44, 45]. Most ovarian cancer cases are currently diagnosed at advanced stages of the disease (III and IV) with tumour recurrence and chemoresistance as the major causes of the treatment failure [16]. Therefore, there is an urgent need for the identification of biomarkers for use in the early diagnosis of ovarian cancer, determining prognosis, and predicting response to therapy [46]. Moreover, it is essential to develop more effective and less toxic targeted therapies for patients diagnosed with ovarian cancer [2, 9, 47].

In the past three decades, abnormal expression and increased activation of members of the HER family have been reported in a wide range of human malignancies and of these EGFR and HER-2 are important therapeutic targets for treatment with several monoclonal antibody based products and different forms of the HER inhibitors in a wide range of cancers. However, clinical trials with the HER inhibitors in patients with ovarian cancer have been disappointing and none of the HER inhibitors has yet been approved for the treatment of patients with ovarian cancer. This may be due to the lack of studies examining the relative expression, cellular location, prognostic significance and predictive value of all members of the HER family proteins in patients with ovarian cancer [48–50]. Indeed, as trans–activation of the HER family members through hetero/homodimerization can contribute to tumourigenesis, and human cancers, including ovarian cancer are heterogeneous in nature, it is considered to be essential to determine the relative expression of all members of the HER family in patients with ovarian cancer [51]. Also the interaction between the HER family members and other tyrosine kinases may be another route for promoting oncogenic signaling pathways and maintaining cancer cell survival and proliferation. Therefore, in this comprehensive study for the first time to our knowledge, we investigated the co-expression and cellular location of all members of the HER-family, as well as the EGFRvIII, c-MET and IGF-1R, and putative CSC marker CD44 in 60 patients with FIGO stages III and IV. We also investigated their impacts on overall survival and disease-free survival.

Several studies examined the expression level of the individual member of the HER family in patients with ovarian cancer. The expression of EGFR, HER-2, HER-3 and HER-4 reported in the literature for ovarian cancer exhibits wide variation ranging from 9–90%, 6.4–52%, 16–69%, and 65–90% of the cases examined respectively, with EGFRvIII expression being rare [52–59]. In this study of 60 FIGO stage III and IV patients, the expression levels of the EGFR and HER-2 were determined by immunohistochemistry using mAb EGFR.113 and mAb 3B5 respectively. At cut off value of >5% of tumours with positive staining, EGFR and HER-2 expression was detected in 62% and 93% of the ovarian cancer cases respectively (Table 2). Of these, the cellular location of EGFR staining was membranous and cytoplasmic in 33% and 28% of the cases examined, whereas HER-2 immunostaining was predominantly cytoplasmic (83.3%) with only 10% of the cases having membranous expression of HER-2 (Table 2). The results of a meta-analysis of 15 studies involving 2471 patients with ovarian cancer for the EGFR expression found positive EGFR immunostaining in 6.2% to 72.6% (median 35%) of tumours, and in 7 studies (63.6%) EGFR expression was found to be predictive of a poorer overall survival [60]. In the same study, the authors also carried out a meta-analysis of 20 studies involving 3055 patients for the HER-2 expression and found positive immunostaining for HER-2 in 5% to 57% (median 18%) of the cases examined, and 8 out of 20 studies (40%) found HER-2 positivity to be of a significant predictor of overall survival in univariate analysis [60]. This wide variation in the expression level of HER family members is common in ovarian cancer and could be due to the use of various techniques, various antibodies employed for the immunohistochemical detection of such receptors, different scoring system, as well as different patient and population size. Unlike EGFR and HER-2, very few studies have been conducted on HER-3 and HER-4 expression in ovarian cancers and in particular the co-expression all four members of the HER family and their prognostic significance [61–63]. Of the 60 stage III and IV ovarian cases examined here, we found 65% and 45% to be HER-3 and HER-4 positive respectively. Interestingly, the predominant location of HER-3 and HER-4 staining were found to be nuclear (67%), and cytoplasmic (43%) respectively (Table 2). Nuclear expression of HER-3 has also been reported in other types of cancer and associated with increased risk of disease progression [64].

Interestingly, 23% of the cases had co-expression of all four members of the HER family and 43%, 23% and 28% of the cases had co-expression of three members of the HER family namely EGFR/HER-2/HER-3, EGFR/HER-3/HER-4 and EGFR/HER-2/HER-4 (Table 3). When examined at different cut off values, only EGFR staining at >50% of tumour cells (HR = 3.57, CI = 1.07–11.85, *p* = 0.038) was associated with a poorer overall survival in univariate analysis and EGFR expression at >5% of tumour cells (HR = 2.83, *p* = 0.019) and its co-expression with HER-2 (HR = 2.83, *p* = 0.019), HER-3 (HR = 2.48, *p* = 0.023), and HER-2/HER-3 (HR = 2.60, *p* = 0.016), were all associated with poorer disease-free survival (Table 4, Figure 2). While, at the cut off value of >5% of tumour cells with positive immunostaining, 48% and 22% the cases were CD44 and c-MET positive respectively, only CD44 immunostaining of 3+ intensity was associated with a poorer overall survival in both univariate and multivariate analysis, and the c-MET/EGFR co-expression (HR = 3.05, *p* = 0.019) was associated with a poorer disease-free survival (Table 4). Interestingly, only one other study to our knowledge, has determined the relative expression of all four HER family members and c-MET in tissue arrays from 202 tumours from ovarian cancer patients (172 FIGO stages I-IV and 30 stages unknown). They found membranous expression of EGFR, HER-2, HER-3, and c-MET in 25%, 35%, 76%, and 96% of the cases examined [65]. HER-4 was found to be positive in 98% of the cases examined, where immunostaining was based upon membrane, cytoplasmic or nuclear staining. They did not find any significant association between the expression levels of these growth factor receptors and PFS in both univariate analysis and multivariate analysis [65]. More recently, Mehner and colleagues reported the result of immunohistochemical staining of EGFR in tissue microarrays from 488 ovarian cancer patients. They found while 90% of their tumor specimens had some EGFR staining and 53% had membranous staining, the EGFR staining alone was not of prognostic value [59]. Also, there is currently conflicting data on the prognostic significance of CD44 in patients with ovarian cancer. While CD44 expression was associated with unfavorable prognostic outcome in FIGO III and IV ovarian cancer in one study [66], Zhang and colleagues reported no correlation between CD44 expression and prognosis in advanced stage ovarian cancer [67]. In another study involving 96 patients with serous ovarian epithelial cancer stages IIB-IVA, 49% of the cases were CD44 positive and this was associated with a statistically significant shorter DFS and overall survival in such patients (HR 6.8, 2–4-19.2 *p* ≤ 0.001) [68]. The expression of aldehyde dehydrogenase (ALDH1), which is another putative ovarian cancer stem cell biomarker, has also been correlated with significantly lower progression free survival and the maintenance of ovarian cancer cell like properties in ovarian cancer patients [69, 70], highlighting the importance of co-targeting of ovarian cancer stem cells in combination with other therapeutics in ovarian cancer. Surprisingly, none of the 60 cases examined in our study was found to be IGF-1R positive. There are currently very few studies of IGF-1R in ovarian cancer patients. While an earlier study reported that of 80 ovarian cancer cases (FIGO I–IV), only 9 (11%) of patients were IGF-1R positive [71], more recent studies have shown that IGF-1R expression was high (>50%) particularly in high-grade ovarian cancer cases [72–74].

In summary, our results suggest that co-expression of two, three or all four HER-family members and CD44 overexpression are common in patients with Stages III and IV ovarian cancers and that EGFR and CD44 expression at different cut off values may be of prognostic value. However, further investigations involving a larger group of ovarian cancer patients are warranted to confirm the prognostic value of co-expression of HER family members in patients with ovarian cancer. Moreover, as the targets for therapeutic interventions with monoclonal antibody-based products and small molecules HER TKIs are the HER family proteins and not HER genes, further examination of tumour specimens from ovarian cancer patients in clinical trials with various types of HER inhibitors are warranted. Such studies would help to determine whether the expression of all four HER proteins would be a more reliable predictive biomarker(s) for stratification of ovarian cancer patients who would benefit from therapy with various types of the HER inhibitors [46].

## MATERIALS AND METHODS

### Patient information

Ethical approval was obtained from the Research and Development Committee of the Royal Surrey County Hospital for examination of tumour specimens from patients with ovarian cancer for use in this study. Sixty patients with FIGO stage III and IV ovarian cancer were included in this retrospective study. All patients had radical surgery and cycles of chemotherapy (paclitaxel, carboplatin and gemcitabine) and 8 patients received anti-VEGF mAb bevacizumab between 2009 and 2013. As only archived tumour specimens were included in this study, the ethics committee waived the need for patient consent and patient records/information were analysed anonymously. Those cases with no follow-up information, and poor or insufficient tumour blocks were excluded from this study.

### Immunohistochemistry

The following primary antibodies were used in this study: mouse anti-EGFR (1:10, Novacastra, UK), mouse anti-HER-2 (1:150, Insight Biotechnology, UK), mouse anti-HER-3 RTJ.2 (1:50, Insight Biotechnology, UK), rabbit anti-HER-4 (1:20, Fisher Scientific, UK), rabbit anti-EGFRvIII (1:150, Bioss Antibodies, UK), mouse anti-IGF-1R (4 ug/mL, Merck Millipore, UK), mouse anti-c-MET (1:500, Novacastra, UK) and mouse anti-CD44 (1:40, DAKO, UK). Formalin-fixed paraffin-embedded (FFPE) sections of tumour specimens (3 μM) were cut in serial sections, and then subjected to antigen retrieval and incubation with primary antibodies as described previously by Khelwatty and colleagues [22]. The optimisation for HER-3 and IGF-1R staining was conducted using the MCF-7 (HER-3+) breast cancer cell line and tumour section of the placenta respectively. Staining of the slides was carried out on a Ventana Benchmark Ultra autostainer with the Ultra View DAB kit (Roche, UK). Following this, all slides were rehydrated and counterstained with haematoxylin, mounted and cover slipped.

### Scoring system

In this study, the immunostaining of the tumour sections were scored based on the percentage of tumour cells that had positive immunostaining at different cut-off values (i.e. >5%, >10%, >20% and >50%) and the intensity of immunostaining (i.e negative 0, weak positive 1+, moderate positive 2+ and strongly positive 3+). The immunostaining was also scored based on the cellular location of the antigen i.e. whether the staining was predominantly present in the membrane, cytoplasm or nucleus of the cells. Two independent trained observers, without prior knowledge of the clinicopathological parameters, conducted the scoring and any disparity in scoring was resolved by simultaneous reassessment of the staining by both observers.

### Statistical analysis

The Chi-Squared test (Pearson Chi-square) and Fisher's exact test were used to investigate the association between immunohistochemistry score and patient clinicopathological data. Kaplan-Meier survival plots and log-rank tests were used to analyse the differences between the groups. The Cox-regression model was used to perform univariate and multivariate analyses. For the multivariate analysis age, FIGO stage, grade and serous subtype were used simultaneously as covariates for both overall survival and disease-free survival, and P≤0.05 was considered statistically significant. All statistical analysis were carried out using the PASW Statistics 23 (SPSS Inc), as described in our previous studies [22].
